# Using Anthropometric Indicator to Identify Hypertension in Adolescents: A Study in Sarawak, Malaysia

**DOI:** 10.1155/2018/6736251

**Published:** 2018-08-01

**Authors:** Whye Lian Cheah, Ching Thon Chang, Helmy Hazmi, Grace Woei Feng Kho

**Affiliations:** ^1^Department of Community Medicine & Public Health, Faculty of Medicine & Health Sciences, Universiti Malaysia Sarawak, Kota Samarahan, Sarawak, Malaysia; ^2^Department of Nursing, Faculty of Medicine & Health Sciences, Universiti Malaysia Sarawak, Kota Samarahan, Sarawak, Malaysia

## Abstract

This cross-sectional study was conducted to determine the predictive power of anthropometric indicators and recommend cutoff points to discriminate hypertension among adolescents in Sarawak, Malaysia. A total of 2461 respondents aged 12-17 years participated in this study with mean age of 14.5±1.50 years. All anthropometric indicators had significant area under the ROC curve, with body mass index (BMI) and waist circumference (WC) ranging from 0.7 to 0.8. The best anthropometric indicators for predicting hypertension for boys were WC, BMI, and waist-to-height ratio (WHtR). For girls, BMI was the best indicators followed by WHtR and WC. The recommended BMI cutoff point for boys was 20 kg/m^2^ and 20.7 kg/m^2^ for girls. For WC, the recommended cutoff point was 67.1 cm for boys and 68.2 cm for girls. BMI and WC indicators were recommended to be used at the school setting where the measurement can easily be conducted.

## 1. Introduction

Adolescent obesity has become a growing health concern, an epidemic that affects both developing and developed countries [1]. The prevalence has reached to the level that warrant an immediate attention on the primary and secondary prevention of overweight and obesity in children and adolescents. It is during the childhood and adolescents period where they develop their eating and activity pattern that can affect their lifestyle in adulthood. With the current nutritional transition that involved availability of fast foods, soft drinks, sedentary lifestyle, physical inactivity, and increase use of technology related gadgets, many adolescents aged 10-19 years were found to be less active and eat more, resulting with increase of body mass index (BMI) and fat [2]. Such unhealthy trends contributed to the increase of comorbidities such as elevated blood cholesterol, type 2 diabetes mellitus, and hypertension [3].

There were many studies that linked hypertension with overweight and obese among the adolescents. Although the evidences gathered have a mixed conclusion on the relationship between hypertension and body fat [4], measurement of body fat using anthropometric indicators had proven to be an effective approach in predicting hypertension, particularly in a large population and community-based studies [5]. Beside the use of BMI in assessing nutritional status, other indicators such as waist circumference (WC), waist-height ratio (WHtR), and conicity index (C index) were other common assessment tools where WC measures the overall body fat, WHtR assess the proportion of central fat by height, and C index measures the abdominal fat.

It is not a common practice to screen for hypertension among adolescents in the community routinely. However, detection of high blood pressure plays an important role in control and prevention of hypertension. However, young people are less likely than older adults to be aware of their risk for hypertension and screen for hypertension on their own. Perhaps one of the reasons is that they may think themselves as invincible and unlikely to be at risk for chronic diseases such as hypertension [6]. Provision of healthcare to this segment of population is often been less emphasized as compared to other age groups.

In Malaysia, the prevalence of prehypertension and hypertension among adolescents was reported to be 11.1% and 11.6%, respectively [7]. Adolescents who are hypertensive tend to be hypertensive in their adult life [3]. The Malaysia National and Health Morbidity Survey (NHMS) 2011 reported that 43.5% of the Malaysian adult population was affected by hypertension [8], an increased trend from previous years (42.6% under NHMS III 2006 and 32.9% under NMHS II 1996) [8–10]. These findings indicated the importance of controlling and preventing of hypertension at a younger age.

Using anthropometric indicators as the preliminary screening tool indirectly help to detect elevated blood pressure at the school environment. As the method does not involve the use of complicated gadget and involvement of specialized skills, the measurement can be taken routinely by the school authorities. If the anthropometric indicators show abnormality results, further assessment of blood pressure with subsequently referral for hospital management can be carried out. Thus, the aim of this study was to determine the prevalence of hypertension and predictive power of anthropometric indicators and recommend cutoff points to discriminate high blood pressure among adolescents.

## 2. Methods

This was a cross-sectional study assessing the blood pressure profile among adolescents in Sarawak, Malaysia. The study was funded by the Fundamental Research Grant Scheme, Ministry of Higher Education Malaysia.

Sarawak is the largest state of Malaysia, located at the Island of Borneo. Based on 2015 census, it has an estimated population of 2,636,000, with more than 40 subethnic groups, each with its own distinct language, culture, and lifestyle. The six major ethnic groups are Iban, Chinese, Malay, Bidayuh, Melanau, and Orang Ulu.

The study population comprised 186 secondary school students aged 13-17 years in Sarawak, with enrolment of 200,130 students, obtained from the Ministry of Education (February 2014). Using the sample size formula for finite populations [11] s= [X2Np(1–p)]÷[d2(N–1)+ X2p(1–p)], where s is required sample size, X is Z-score for 99% confidence interval (2.58), N is population size (200,310), p is population proportion (assumed to be 0.5 since this would provide the maximum sample size), d is degree of accuracy or margin of error (0.028), and minimum sample size needed was 2100. A quota of 18 schools were decided for each state and systematic sampling was employed in the selection of schools based on the size of enrolment as well as stratification by urban-rural location. In each selected school, one class was randomly selected for each level of schooling from secondary one to secondary six. The inclusion criteria were those respondents without physical and mentally disability, prediagnosed hypertension, or any illness that could lead to secondary hypertension.

Data collection was carried out by a team of trained field personnel. Anthropometric measurement was done using SECA body meter and portable weighing scale. Respondents were weighed with light clothing without footwear. When measuring height, the respondents were to stand upright barefooted on a flat surface with their back of the heels and occiput against the equipment. Measurement of height was to the nearest 0.1 cm. The weight was recorded to the nearest 0.1 kg. Assessment of body mass index was based on WHO 2007 reference using indicators such as thinness, normal, overweight, and obese [12].

Waist circumference (WC) was measured using a plastic nonelastic tape at the midpoint between the last rib and top of hip bone (iliac crest). The respondents were asked to relax their abdomen and stand upright. The cutoff points for abdominal obesity were based on >90 cm for men and >80cm for women [13].

The waist-to-height ratio (WHtR) was calculated based on WC (cm) by height (cm). As for conicity index (C index), the calculation was based on the following equation [14]:

(1) $$\begin{array}{c} CI=\frac{Waist\;\;Circumference\left(m\right)}{0.109\times \sqrt{Body\;\;Weight\left(kg\right)/Height\left(m\right)}} \end{array}$$

Blood pressure was taken using a digital blood pressure monitor, calibrated with auscultation (a mercury sphygmomanometer) with the correct cuff size for arm circumference. Respondents were asked to rest for 5 minutes and check for no intake of caffeine or medication or no exercise before measurement. Two measurements with an interval of one minute were taken. In the case when the differences of the two readings were above 5 mm Hg or the respondent was found to be in the prehypertension or hypertension level, a third reading was taken. The final reading would be based on the average of all readings taken [15]. Classification of hypertension is based on the Fourth Report on the Diagnosis, Evaluation, and Treatment of High Blood Pressure in Children and Adolescents 2004 [16], where BP is less than the 90th percentile for age, gender, and height; it is classified as normal. BP that falls within 90th to just below 95th percentile is categorized as prehypertension or high-normal.

Statistical analyses were performed using IBM Statistical Package for Social Sciences (SPSS) version 22. Descriptive and inferential statistical analysis was performed based on confidence interval of 95% and p-value of less than 0.05 was regarded as significant. For inferential analysis, prehypertension is regrouped into hypertension, resulting in dichotomous dependent variables, normotensive and hypertensive. The predictive power of anthropometric indicators for hypertension was determined by Receiver Operating Characteristic curves (ROC). The area under ROC curve is used to determine the discriminating power of the anthropometric indicators on hypertension in adolescents. The larger the area under ROC curve, the better the discriminating power of the indicators. This study has adopted 60% as the acceptable value for sensitivity and specificity value, based on the rule of thumbs by Maroco [17] where sensitivity and specificity value between 50% and 80% are considered as acceptable. Using the Youden Index (J) method, the optimal cut point was determined based on the difference between true positive rate and false positive rate over all possible cut-point value [18].

Ethical approval was obtained from the Medical and Ethical Committee of Universiti Malaysia Sarawak (UNIMAS/TNC (AA)-03.02/06-11 Jld.3(1) and Ministry of Education Malaysia. Informed consent was obtained from the parents/caregivers of the respondents prior to the day of data collection. A research information sheet was given to each of the respondents as well as their parents/caregivers. The respondents were also being briefed the research information on the day of the data collection. Respondents were ensured of the confidentiality of their data and they have the right to withdraw from the study at any time.

## 3. Results

A total of 2540 respondents have consented to participate in this study. However, 79 (3.1%) were excluded due to absent from school on the day of data collection and a number have refused despite parental consent, so the final sample size was 2461 (96.9% response rate). There were more girls than boys (58% versus 42%), mean age of 14.5±1.50 years, with majority from Iban and Malay ethnic groups. The mean weight, height, and waist circumference were higher among boys compared to girls, while BMI and WHtR were higher among girls. However, the gender differences were found significant only in weight, height, and WHtR. Both gender has similar C index. There were more boys than girls who were found to be prehypertensive and hypertensive and this difference was significant. More girls were found to be overweight than boys; however, in the obese category, the reverse was true. The detailed information is presented in Table 1.

BMI showed an increasing trend among respondents, especially age group 13 years and above. Waist circumference and systolic and diastolic blood pressure also showed similar pattern but were not consistently increased from younger to older age groups (refer Table 2).

**Figure 1:**
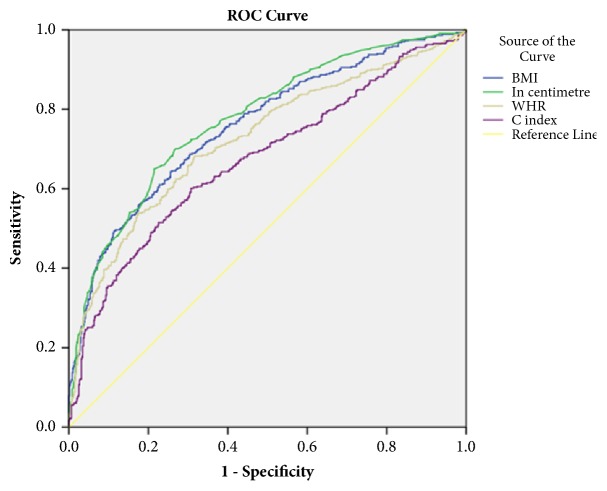
ROC curves that compare four anthropometric indicators of obesity as discriminators of hypertension (boys). BMI: body mass index, in centimeter-waist circumference; WHR: waist-height ratio; C index: conicity index.

**Figure 2:**
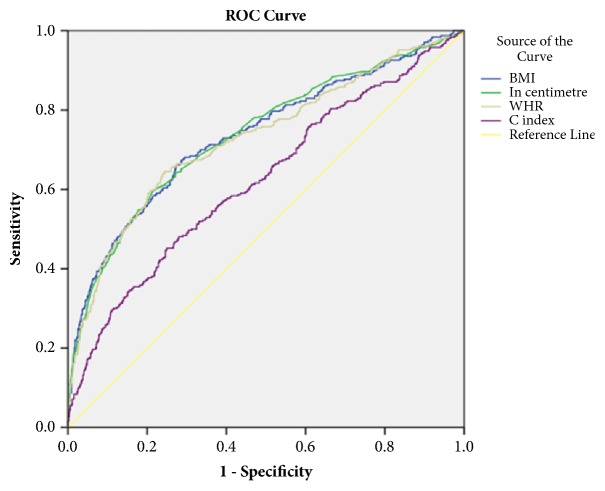
ROC curves that compare four anthropometric indicators of obesity as discriminators of hypertension (girls). BMI: body mass index, in centimeter-waist circumference; WHR: waist-height ratio; C index: conicity index.

Table 3 and Figures 1 and 2 show the final models using Stepwise logistic regression analyses and ROC analyses. All the anthropometric indicators showed significant association with hypertension for both logistic regression and ROC curve, with BMI and WC taking over larger area and fair readings (0.7-0.8). Based on cutoff point, sensitivity, and specificity results, the preferred anthropometric indicator for predicting hypertension for boys was WC and BMI, followed by WHtR. However, for girls, BMI was the best indicator followed by WHtR and WC.

## 4. Discussion

The prevalence of hypertension in this study was 22.5% for males and 12.9% for females. In terms of prehypertension, the prevalence was found to be slightly lower with 19.3% for males and 8.8% for females. Our results showed higher prevalence compared with other local studies [7], indicating that in a short span of five years the prevalence of prehypertension and hypertension has increased although the study location was different but it was a representative of Malaysia. Another finding of this study also indicated that the prevalence of overweight and obese is also high among males (overweight 11.7%, obese 13.7%) and females (overweight 12.7%, obese 10.7%) which might be closely related to the high prevalence of hypertension. Such observation strongly suggested an immediate attention to address this health issues which may lead to hypertension and obesity related health complication in the later life.

One of the purposes of this study was to determine the predictive power and establish the cutoff points of anthropometric indicators for the prediction of hypertension. It is hoped that with this finding a routine health checkup using anthropometric measurement at the school level can be routinely implemented to screen and detect adolescents with signs of hypertension for further investigation at the clinical setting.

Among all the anthropometric indicators, BMI and WC had the highest sensitivity and specificity value, supporting the evidence from previous study that both indicators were equally well related to hypertension regardless of age and gender [19]. However, in one study, such findings were argued where central obesity measured by WC was perceived as a stronger predictor in hypertension. This is supported by a big population study in China where the findings based on 500,000 adults reported that central obesity was the main predictor [20]. In adolescent studies, the evidences regarding anthropometric indicators with hypertension in children and adolescents are inconclusive. Some studies showed that BMI and WC were significant predictors for hypertension with OR 2.60 and 1.85, respectively [21]. While one study found that WHtR and WC are better predictors than BMI for hypertension, another study would exclude WHtR and supported BMI and WC [22, 23].

According to Sardinha et al. [24], such findings need to be interpreted with cautious. This is because the magnitude of the association between anthropometric indicators and cardiometabolic risk was stronger in overweight and obese children and adolescents compared to their counterpart. In their study, they found that being overweight was associated with more than twofold increased odd of having clustered cardiometabolic risk in adolescents while being obese was associated with more than 14 times increased risk compared to their normal weight samples. They concluded that although BMI, WC, and WHtR may not be strong predictors of cardiometabolic risk, at higher level of adiposity, the cluster of cardiovascular risk factors increases as BMI, WC, or WHtR increases.

Consistent with literatures, C index in this study presented a lower predicting power, sensitivity, and specificity as compared to other anthropometric indicators. Cutoff point for this indicator was 1.12, similar to other studies ranging from 1.13 to 1.23 [25, 26]. The sensitivity and specificity were lower as compared to literatures (ranging from 57.4 to 64.3%) [25, 26]. The evidences on C index and its relationship with hypertension was limited; however studies among risk of diabetes and hypertension in women showed that there was an association between C index with HDL cholesterol [27].

In terms of cutoff point for WC, the current study showed that girls had higher WC as compared with boys, consistent with other studies [25, 28]. The results also indicated a lower WC for both boys and girls compared to literatures, where most of the WCs ranged from 70 cm or more for boys and 80 cm or more for girls. One possible explanation was the body frame size for Asians adolescents is smaller than those in the western population. Based on the Malaysian waist circumference percentile curves for children and adolescents aged 6 to 16.9 years [29], the cutoff point for boys was close to the 50th percentile and the girls was close to 75th percentile.

For WHtR, the cutoff point for boys was lower than girls with 0.42 and 0.44, respectively, consistent with others studies [25, 30]. In one study among adult females, WHtR indicator was ranked with the highest discriminatory power in hypertension risk screening. It was further argued that accumulation of body fat at the abdominal part maximize the hemodynamic changes that caused endothelial dysfunction and dysregulation of hepatic metabolism that eventually promote the onset of cardiovascular disorders [31]. In additional to that, WHtR incorporated the measurement of height in its formula which proved to be more accurate in determining hypertension risk [32].

Unlike past studies on the cutoff point for BMI, this study showed higher BMI cutoff point for girls (20.7 kg/m2) as compared to boys (20.0 kg/m2). In Iran, the mean BMI for predicting hypertension among boys was higher than this study (21.9 kg/m2), but its cutoff point for girls was lower (19.1 kg/m2) [30]. Although BMI is not as accurate in differentiating distribution of fat in different body compartments as compared to WC and WHtR, it has served as the most common used anthropometric indicator in screening cardiovascular risk for children and adolescents. Perhaps a reclassification of BMI in screening for hypertension should be further explored.

Because the current study was a cross-sectional, therefore the causality association cannot be affirmed. Besides that, although the protocol of conducting the blood pressure was based on an interval of one minute [16], there might be an overestimation of the prevalence of hypertension that resulted higher sensitivity. Nevertheless, this is a common issue related to the use of screening tools at the community level.

## 5. Conclusion

In conclusion, based on the findings, it is recommended to use BMI and WC indicators in detecting hypertension among adolescents in Sarawak. A cutoff point of 20 kg/m^2^ for boys and 20.7 kg/m^2^ for girls was recommended for BMI, and a cutoff point of 67.1 cm for boys and 68.2 cm for girls was recommended for WC to detect elevated blood pressure. Both indicators can be used at the school setting where scale, stadiometer, and tape measure can easily be made available. Those adolescents who are identified can be further referred for clinical investigation and subsequently go for treatment and intervention.

## Figures and Tables

**Table 1:** Sociodemographic and health profile among male and female respondents (N=2461).

Variables	mean±SD / n(%)		*p* value
	Male (n=1033)	Female (n=1428)	
Age (years)	14.4 ± 1.48	14.5 ± 1.51	0.04
Weight (kg)	55.5 ± 14.78	51.0 ± 12.80	<0.001*∗∗*
Height (m)	1.6 ± 0.08	1.5 ± 0.06	<0.001*∗∗*
BMI (kg/m^2^)	21.3 ± 4.72	21.6 ± 4.78	0.08
WC (cm)	71.3 ± 11.56	70.2 ± 18.46	0.106
WHtR	0.4 ±0.07	0.5 ± 0.11	<0.001*∗∗*
C Index	1.1±0.07	1.1±0.27	0.654
Ethnicity			0.223
Iban	308 (29.8)	429 (30.0)	
Malay	301 (29.1)	380 (26.6)	
Bidayuh	116 (11.2)	140 (9.8)	
Chinese	181 (17.5)	294 (20.6)	
Others	127 (12.4)	185 (13.0)	
Blood pressure			<0.001*∗∗*
Normal	602 (58.3)	1118 (78.3)	
Pre-hypertension	199 (19.3)	125 (8.8)	
Hypertension	232 (22.5)	185 (12.9)	
BMI			0.004*∗∗*
Thinness	78 (7.6)	71 (5.0)	
Normal	692 (67.0)	1022 (71.6)	
Overweight	121 (11.7)	182 (12.7)	
Obese	142 (13.7)	153 (10.7)	

*∗∗* Significant at p<0.001; BMI: body mass index; WC: waist circumference; WHtR: waist-to-height ratio; C Index: conicity index.

**Table 2:** Anthropometric measurement and blood pressure according to age among male and female respondents (N=2461).

	Sex	Age (year±SD)						*p* value
		12 years	13 years	14 years	15 years	16 years	17 years	
		M=106; F=149	M=240; F=292	M=215; F=261	M=180; F=281	M=214; F=308	M=78; F=137	
BMI	M	21.09±5.30	20.80±4.97	20.96±5.03	21.57±4.21	21.78±4.46	21.85±3.89	0.161
	F	21.38±5.62	20.55±4.27*∗*	20.86±4.10	21.96±4.86*∗*	22.76±5.06*∗*	22.32±4.56*∗*	<0.01
WC	M	70.42±13.30	69.77±12.59	70.66±12.09	71.80±10.08	72.92±10.33	72.81±10.03	0.045
	F	73.73±49.48*∗*	67.68±9.20*∗*	68.58±8.62	70.21±9.71	72.04±10.70	70.75±9.82	0.006
WHt	M	0.46±0.08	0.45±0.07	0.44±0.07	0.44±0.06	0.44±0.06	0.44±0.06	0.070
	F	0.49±0.31*∗*	0.45±0.06*∗*	0.45±0.05*∗*	0.46±0.06	0.46±0.07	0.46±0.06	0.01
C Index	M	1.14±0.08*∗*	1.12±0.07	1.12±0.07	1.11±0.06*∗*	1.12±0.06*∗*	1.11±0.06	0.006
	F	1.20±0.81*∗*	1.12±0.06*∗*	1.12±0.07	1.11±0.06*∗*	1.12±0.06*∗*	1.11±0.06	0.03
SBP	M	112.35±13.18*∗*	116.23±13.77	118.66±14.36*∗*	119.34±13.09*∗*	120.59±12.96*∗*	120.54±13.90*∗*	<0.01
	F	112.41±14.30	109.39±11.23*∗*	109.93±11.40	111.79±10.81	112.29±12.58*∗*	111.69±13.11	0.013
DBP	M	63.12±10.19*∗*	63.89±9.50*∗*	65.15±10.23	64.73±9.27	66.92±9.58*∗*	65.17±7.88	0.007
	F	65.66±9.90	64.62±8.61*∗*	65.25±9.02	66.40±7.50	67.04±9.90*∗*	66.84±8.87	0.011

M: male; F: female.

*∗*One-way analysis of variance was applied to see the differences between age group and p<0.05 was considered as statistically significant.

**Table 3:** Age-adjusted Logistic regression model and discriminative capability for hypertension by sex (N=2461).

	Male				Female			
	BMI	WC	WHtR	C Index	BMI	WC	WHtR	C Index
**Logistic Model**								
Constant	-6.605	-8.670	-9.448	-15.021	-4.712	-7.288	-7.803	-10.632
Exp (B)	1.278	1.111	2.778	5.114	1.223	1.099	2.167	2.588
*p* value	<0.01	<0.01	<0.01	<0.01	<0.01	<0.01	<0.01	<0.01
**Discriminative Capability**								
Area under ROC curve	0.76 (0.73-0.79)	0.76 (0.73-0.79)	0.76 (0.73-0.79)	0.76 (0.73-0.79)	0.74 (0.70-0.77)	0.74 (0.70-0.77)	0.74 (0.70-0.77)	0.74 (0.70-0.77)
Cutoff point	20.0	67.1	0.42	1.11	20.7	68.2	0.44	1.12
Sensitivity	75.4	77.3	71.2	64.3	72.9	71.3	71.9	57.4
Specificity	60.3	61.8	60.5	60.0	60.0	61.6	60.0	60.0

## Data Availability

This project is funded by Ministry of Higher Education. Therefore data can only be made available with application to the sponsor.
